# Efficient Similarity Point Set Registration by Transformation Decomposition

**DOI:** 10.3390/s20154103

**Published:** 2020-07-23

**Authors:** Chen Wang, Xinrong Chen, Manning Wang

**Affiliations:** 1 Digital Medical Research Center, School of Basic Medical Sciences, Fudan University, Shanghai 200032, China; wangchen17@fudan.edu.cn; 2 Shanghai Key Laboratory of Medical Imaging Computing and Computer Assisted Intervention, Shanghai 200032, China; 3 Academy for Engineering and Technology, Fudan University, Shanghai 200433, China; chenxinrong@fudan.edu.cn

**Keywords:** branch and bound, similarity point set registration, transformation decomposition

## Abstract

Point set registration is one of the basic problems in computer vision. When the overlap ratio between point sets is small or the relative transformation is large, local methods cannot guarantee the accuracy. However, the time complexity of the branch and bound (BnB) optimization used in most existing global methods is exponential in the dimensionality of parameter space. Therefore, seven-Degrees of Freedom (7-DoF) similarity transformation is a big challenge for BnB. In this paper, a novel rotation and scale invariant feature is introduced to decouple the optimization of translation, rotation, and scale in similarity point set registration, so that BnB optimization can be done in two lower dimensional spaces. With the transformation decomposition, the translation is first estimated and then the rotation is optimized by maximizing a robust objective function defined on consensus set. Finally, the scale is estimated according to the potential correspondences in the obtained consensus set. Experiments on synthetic data and clinical data show that our method is approximately two orders of magnitude faster than the state-of-the-art global method and more accurate than a typical local method. When the outlier ratio with respect to the inliers is up to 1.0, our method still achieves accurate registration.

## 1. Introduction

Point set registration is a fundamental problem in computer vision. It is widely used in three-dimensional reconstruction [1,2], medical image analysis [3,4,5], mobile robots [6,7] and autonomous driving [8]. The goal of point set registration is to estimate a transformation to align two point sets, namely moving point set and reference point set. This paper focuses on the scenarios of estimating a similarity transformation between two 3D point sets.

A large body of methods have been proposed to solve the three-dimensional rigid point set registration problem [9,10]. Similarity point set registration has only increased a scale factor compared to rigid registration, so many studies have extended the rigid registration method to estimate similarity transformation. The iterative closest point (ICP) [11] is the most typical rigid registration method; it has been extended for similarity point set registration in many researches [12,13,14,15,16,17,18]. However, the optimization scheme of ICP is the expectation maximization (EM) [19] type so it can only converge to a local optimum. Another line of research represents each point set with a probability density and registers the two point sets by aligning the two probability densities [20,21,22]. Some methods use similar ideas to achieve non-rigid registration between two point sets [23]. Though these non-rigid point set registration methods can also be used for similarity registration, these methods can also only converge to a local optimum.

In recent years, some global point set registration methods have emerged, such as Go-ICP [24], GOGMA [25] and so on [26,27,28,29]. These methods parameterize rigid point set registration with the rigid transformation, which consists of a three-Degrees of Freedom (3-DoF) rotation and 3-DoF translation between the two point sets to be registered, but they cannot be simply extended to estimate 7-DoF similarity transformation, which consists of another DoF scale between the two point sets. In addition, most of these global methods use branch and bound (BnB) optimization framework, but the time complexity of BnB optimization is exponential in the dimensionality of the problem. It is already very slow for these global methods to perform 6-DoF rigid registration, and it will be very inefficient for them to do 7-DoF similarity registration. Asymmetric point matching (APM) [30] parameterizes point set registration with the correspondence between points from each set and develops a deterministic global method to solve affine point set registration problem. APM can also be used to solve the similarity point set registration problem, but it assumes a point-to-point correspondence, which makes it difficult to register partially overlapping point sets or point sets with outliers.

In this paper, we propose a global similarity point set registration method by using BnB optimization framework. To avoid the inefficiency of the BnB-based method in solving high-dimensional problems, we introduce a transformation decomposition approach so that the translation, rotation and scale can be estimated separately. Two BnB-based algorithms are used to globally estimate the 3-DoF translation and the 3-DoF rotation, and the two algorithms are fast because the problem dimensionality is low. To the best of our knowledge, this is the first global similarity point set registration method. In addition, a consensus set-based objective function is used for the translation and rotation estimation, so the proposed method is robust to outlier and partial-overlap. Extensive experiments show that the proposed method is approximately two order of magnitudes faster than state-of-the-art global method and much more accurate than local methods in similarity point set registration.

The rest of this paper is organized as follows. In Section 2, the related work is reviewed. The proposed method is introduced in Section 3. In Section 4, results on synthetic and clinical data are presented and discussed. Finally, conclusions and limitations are discussed in Section 5.

## 2. Related Work

We review the works on the following three topics that are related to this paper: local methods for similarity point set registration, global point set registration methods and transformation decomposition method developed in point set registration.

ICP is the most widely used local point set registration method. ICP first determines the correspondence using an initial guess of transformation between the two point sets to be registered and then iterates between updating transformation and determining correspondences. ICP was originally developed for rigid point set registration, and many works have been done to extend it for similarity point set registration. For example, Zha et al. [18] used extended feature images to establish accurate correspondence, and then integrated the scale factor into an improved ICP algorithm to achieve precise image registration. Du et al. [31] proposed an objective function based on bidirectional distance, introducing overlap ratio and scale factor. Furthermore, a new isotropic scale ICP algorithm is proposed, which can automatically calculate the scale transformation, correspondence and overlap ratio of each iteration. Although this method is very robust, it is time-consuming to establish a bidirectional correspondence. To speed up the isotropic scaling registration, Li et al. [16] introduced a sparse-to-dense hierarchical model in ICP. Ying et al. [17] proposed the Scale-ICP method. By adding a scale factor into ICP, the registration problem was transformed into a constraint optimization problem on a seven-dimensional nonlinear space, and then the singular value decomposition method was used for iterative solution. However, Scale-ICP may be affected by local dissimilarity, so Du et al. [15] added the corner point constraint to the objective function and proposed a new isotropic scaling ICP algorithm. Wu et al. [13] developed a robust scale ICP algorithm by using correntropy [32] to substitute the mean square error (MSE) as the new similarity measure. Chen et al. [12] proposed a robust algorithm based on correntropy and ICP. Yang et al. [14] combined the kernel mean *p*-power error (KMPE) [33] loss measure with ICP framework to model the similarity and affine registrations. The optimization scheme of ICP is the EM [19] type so it can only converge to a local optimum. The similarity point set registration methods extended from ICP also have this drawback. To relieve the problem of local convergence, some researches model point sets as probability densities and achieve point set registration by aligning the two probability densities, such as the GMMReg method proposed by Jian et al. [22], in which probability density is constructed by using Gaussian mixture model (GMM). Some other methods also use this idea to achieve non-rigid point set registration and they can also be used for similarity point set registration. A typical example of them is the coherent point drift (CPD) method proposed by Myronenko et al. [23]. The objective functions of these methods can be made smoother than that of ICP, so that they can have a larger basin of convergence, but a good initialization is still needed for them to achieve an accurate registration.

In recent years, there has been a series of work utilizing BnB to globally estimate the rigid transformation between two point sets, such as Go-ICP [24], GOGMA [25] and so on [34,35,36]. However, these methods focus on rigid point set registration and they cannot be simply extended to solve the 7-DoF similarity registration problem. In addition, the time complexity of BnB is exponential in the dimensionality of the problem. It is already very slow to estimate the 6-DoF rigid transformation, and it will be even slower to use them to estimate the 7-DoF similarity transformation. APM [30] is a global affine point set registration method, and it can be used to globally solve the similarity point set registration problem. In APM, the objective function of registration is defined on transformation and point corresponding matrix, and it assumes a point-to-point correspondence to achieve a theoretically global optimal solution, which makes it difficult to register partially overlapping point sets or point sets with outliers. Li et al. [37] proposed an global affine point set registration method based on matching probability density and BnB. Though this method can also be used for similarity point set registration, it estimates the affine directly and the computation complexity of estimating a 12-DoF affine transformation is prohibitive.

An effective way of speeding up the BnB based global point set registration method is to decompose the transformation into components of lower dimensionality. For example, a rigid transformation in SE(3) can be decomposed into a rotation in SO(3) and a translation in $R^{3}$. Here SE(3) and SO(3) are special Euclidean group and special orthogonal group in three dimensions, respectively, and they are the parameter space of 3D rigid transformation and 3D rotation [38]. Two studies utilize the idea of transformation decomposition for global rigid point set registration. Straub et al. [27] constructed a surface normal distribution from the original point set, which is translation invariant, and used BnB to optimize rotation to align the two surface normal distributions first. Then it applied the obtained optimal rotation to the original point set, and used another BnB to optimize translation. Although this method decoupled the six-dimensional parameter space into two three-dimensional parameter spaces, it was time-consuming to construct translation-invariant features and required GPU acceleration. Liu et al. [28] proposed a rotation invariant feature to decompose rigid transformation, but it cannot be used for similarity registration. Yang et al. [39,40] estimated scale, rotation and translation separately and proposed a polynomial time method, but putative correspondences between the two point sets to be registered are needed.

In this paper, we propose an efficient global method for similarity point set registration, in which the similarity transformation is decomposed into translation, rotation, and scale, and the three parameters are estimated sequentially. The transformation decomposition makes the registration very efficient. Concretely, our contribution in this paper includes two aspects:

- We propose a rotation and scale invariant feature (RSIF) utilizing the angle invariance in similarity transformation. Using this RSIF, we can first globally search for the translation between the two point sets to be registered. A BnB-based global optimal translation search algorithm is developed to match the RSIF sets constructed from the two original point sets.
- Then we propose a globally optimal rotation search algorithm, which is not influenced by the relative scale, to estimate the optimal rotation between the two original point sets after applying the relative translation obtained in the previous step. Finally, the scale is estimated according to the potential correspondences obtained in rotation estimation.

## 3. Method

Suppose there is a similarity transformation between the moving point set $X={\left\{x_{i}\right\}}_{i=1}^{X}$ and the reference point set $Y={\left\{y_{j}\right\}}_{j=1}^{Y}$. For a pair of corresponding points $y_{j}\in Y$ and $x_{i}\in X$, we have

(1) $$y_{j}={sR}^{*}\left(x_{i}+t^{*}\right)$$

where $t^{*}\in R^{3}$ and $R^{*}\in SO\left(3\right)$ are the translation and rotation from X to Y, respectively, and *s* is the scale.

Most existing BnB-based global methods jointly search every parameter. In this scenario, the 7-DoF similarity transformation is a big challenge for BnB, because the time complexity of BnB optimization is exponential in the dimensionality of the problem. In this paper, an efficient similarity point set registration method based on transformation decomposition is proposed. The RSIF is first introduced to decouple similarity transformation in Section 3.1. Then in Section 3.2, the BnB-based global optimal translation search algorithm is given to align the RSIF sets generated from the two original point sets to be registered. In Section 3.3, the complete similarity point set registration algorithm is given.

### 3.1. Rotation and Scale Invariant Feature

For a set of three points $\left\{x_{i1},x_{i2},x_{i3}\right\}$ from the moving point set, a triple $\left(\begin{array}{c} ∠(x_{i1},x_{i2})\\ ∠(x_{i1},x_{i3})\\ ∠(x_{i2},x_{i3}) \end{array}\right)$ is constructed, where $∠\left(•,•\right)$ denotes the angular distance between two vectors. This triple is invariant with respect to scale and rotation of $x_{i1},x_{i2}$ and $x_{i3}$ around the origin. Thus,

(2) $$\left(\begin{array}{c} ∠(s{\mathrm{Rx}}_{i1},s{\mathrm{Rx}}_{i2})\\ ∠(s{\mathrm{Rx}}_{i1},s{\mathrm{Rx}}_{i3})\\ ∠(s{\mathrm{Rx}}_{i2},s{\mathrm{Rx}}_{i3}) \end{array}\right)=\left(\begin{array}{c} ∠(x_{i1},x_{i2})\\ ∠(x_{i1},x_{i3})\\ ∠(x_{i2},x_{i3}) \end{array}\right)$$

where R∈SO(3) and s∈R. This triple is called a RSIF.

Let $p_{i1,i2,i3}=\left(\begin{array}{c} ∠(x_{i1},x_{i2})\\ ∠(x_{i1},x_{i3})\\ ∠(x_{i2},x_{i3}) \end{array}\right)$ denote the RSIF constructed from a set of moving points $\left\{x_{i1},x_{i2},x_{i3}\right\}$ and $q_{j1,j2,j3}=\left(\begin{array}{c} ∠(y_{j1},y_{j2})\\ ∠(y_{j1},y_{j3})\\ ∠(y_{j2},y_{j3}) \end{array}\right)$ denote the RSIF constructed from a set of reference points $\left\{y_{j1},y_{j2},y_{j3}\right\}$. According to (1) and (2),

(3) $$\begin{array}{c} q_{j1,j2,j3}=\left(\begin{array}{c} ∠(y_{j1},y_{j2})\\ ∠(y_{j1},y_{j3})\\ ∠(y_{j2},y_{j3}) \end{array}\right)=\left(\begin{array}{c} ∠(sR\left(x_{i1}+t\right),sR\left(x_{i2}+t\right))\\ ∠(sR\left(x_{i1}+t\right),sR\left(x_{i3}+t\right))\\ ∠(sR\left(x_{i2}+t\right),sR\left(x_{i3}+t\right)) \end{array}\right)=\left(\begin{array}{c} ∠(x_{i1}+t,x_{i2}+t)\\ ∠(x_{i1}+t,x_{i3}+t)\\ ∠(x_{i2}+t,x_{i3}+t) \end{array}\right):=F\left(p_{i1,i2,i3},t\right) \end{array}$$

A function F(p,t) is defined to express that the two RSIFs are related by the translation t.

$P={\left\{p^{m}\right\}}_{m=1}^{M}$ is used to denote the set of RSIFs constructed from point sets $\left\{x_{i1},x_{i2},x_{i3}\right\}$, where $x_{i1},x_{i2},x_{i3}\in X$ and i1≠i2≠i3. $Q={\left\{q^{n}\right\}}_{n=1}^{N}$ is used to denote the set of RSIFs constructed from point sets $\left\{y_{j1},y_{j2},y_{j3}\right\}$, where $y_{j1},y_{j2},y_{j3}\in Y$ and j1≠j2≠j3. The problem of finding the optimal translation from X to Y is changed to be finding the optimal translation from P to Q. In practice, the cardinalities of P and Q are very large, so we match a subset of each of them as $P^{\prime }={\left\{p^{m^{\prime }}\right\}}_{m^{\prime }=1}^{M^{\prime }}$ and $Q^{\prime }{=\left\{q^{n^{\prime }}\right\}}_{n^{\prime }=1}^{N^{\prime }}$. The steps to obtain the subsets to match are as follows.

- All the three-point combinations are screened from moving and reference point set. Each three-point combination can construct a RSIF. This paper chooses 300 RSIFs with the largest angular distance, and denotes them as $P_{1}$ and $Q_{1}$.
- For $p_{i1,i2,i3}=\left(\begin{array}{c} ∠(x_{i1},x_{i2})\\ ∠(x_{i1},x_{i3})\\ ∠(x_{i2},x_{i3}) \end{array}\right)$, it is constructed from a set $\left\{x_{i1},x_{i2},x_{i3}\right\}$. Take the difference between these three vectors and obtain a new vector $g_{i1,i2,i3}=\left(\begin{array}{c} x_{i1}-x_{i2}\\ x_{i1}-x_{i3}\\ x_{i2}-x_{i3} \end{array}\right)$. Take the angular distance for every two dimensions of $g_{i1,i2,i3}$, and we have $g_{i1,i2,i3}^{\prime }=\left(\begin{array}{c} ∠(x_{i1}-x_{i2},x_{i1}-x_{i3})\\ ∠(x_{i1}-x_{i2},x_{i2}-x_{i3})\\ ∠(x_{i1}-x_{i3},x_{i2}-x_{i3}) \end{array}\right)$.
- Without loss of generality, it assumes that $q_{j1,j2,j3}=\left(\begin{array}{c} ∠(y_{j1},y_{j2})\\ ∠(y_{j1},y_{j3})\\ ∠(y_{j2},y_{j3}) \end{array}\right)$ is the corresponding RSIF of $p_{i1,i2,i3}$. Following the previous step, we have $k_{j1,j2,j3}=\left(\begin{array}{c} y_{j1}-y_{j2}\\ y_{j1}-y_{j3}\\ y_{j2}-y_{j3} \end{array}\right)$ and $k_{j1,j2,j3}^{\prime }=\left(\begin{array}{c} ∠(y_{j1}-y_{j2},y_{j1}-y_{j3})\\ ∠(y_{j1}-y_{j2},y_{j2}-y_{j3})\\ ∠(y_{j1}-y_{j3},y_{j2}-y_{j3}) \end{array}\right)=\left(\begin{array}{c} ∠(sR\left(x_{i1}+t\right)-sR\left(x_{i2}+t\right),sR\left(x_{i1}+t\right)-sR\left(x_{i3}+t\right))\\ ∠(sR\left(x_{i1}+t\right)-sR\left(x_{i2}+t\right),sR\left(x_{i2}+t\right)-sR\left(x_{i3}+t\right))\\ ∠(sR\left(x_{i1}+t\right)-sR\left(x_{i3}+t\right),sR\left(x_{i2}+t\right)-sR\left(x_{i3}+t\right)) \end{array}\right)=\left(\begin{array}{c} ∠(x_{i1}-x_{i2},x_{i1}-x_{i3})\\ ∠(x_{i1}-x_{i2},x_{i2}-x_{i3})\\ ∠(x_{i1}-x_{i3},x_{i2}-x_{i3}) \end{array}\right)$.
- According to the previous steps, $g_{i1,i2,i3}^{\prime }$ and $k_{j1,j2,j3}^{\prime }$ should be equal when the data is clean, and the Euclidean distance between them is close to 0, if there exists noise in data.
- Finally, all the RSIFs that satisfy the condition $∥g_{i1,i2,i3}^{\prime }-k_{j1,j2,j3}^{\prime }\leq \varepsilon_{f}∥$ are chosen from $P_{1}$ and $Q_{1}$, and denoted as $P^{\prime }$ and $Q^{\prime }$, respectively.

On the basis of the consensus set, our objective function of similarity transformation is defined by

(4) $$E_{t}\left(t\right)=\sum_{m^{\prime }}\max_{n^{\prime }}\lfloor ∥F\left(p^{m^{\prime }},t\right)-q^{n^{\prime }}∥\leq \varepsilon_{t}\rfloor$$

where $\left\lfloor •\right\rfloor$ is an indicator function that returns 1 if the inner condition is true and 0 otherwise, ∥•∥ denotes the Euclidean norm in $R^{3}$, and $\varepsilon_{t}$ is the translation search inlier threshold. The optimal translation is obtained by maximizing $E_{t}\left(t\right)$.

### 3.2. Global Translation Search

When BnB-based algorithm is used to globally solve the maximization problem (4), the key is to find a way to calculate the upper bound of $E_{t}$ in a branch T of the parameter space of translation, which means we need to find a function $\bar{E_{t}\left(T\right)}$ satisfying

(5) $$\bar{E_{t}\left(T\right)}\geq \sum_{m^{\prime }}\max_{n^{\prime }}\lfloor ∥F\left(p^{m^{\prime }},t\right)-q^{n^{\prime }}∥\leq \varepsilon_{t}\rfloor ,t\in T$$

Define $D\left(t\right)=∥F\left(p^{m^{\prime }},t\right)-q^{n^{\prime }}∥$ and if it is possible to find a lower bound function

(6) $$\underline{D(T)}\leq D\left(t\right),\forall t\in T$$

then use $\bar{E_{t}\left(T\right)}=\sum_{m^{\prime }}\max_{n^{\prime }}\left\lfloor \underline{D(T)}\leq \varepsilon_{t}\right\rfloor$ as the upper bound of $E_{t}$ in the branch T. Therefore, the problem becomes how to find $\underline{D(T)}$ given a translation cube T. Here, this problem is addressed by natural interval extension [41,42], in which we need to first calculate the bound of each element of $F(p^{m^{\prime }},t)$ when t∈T. Since $F(p^{m^{\prime }},t)$ is computed identically for each dimension, the bounds used in this paper are described using the first dimension as an example.

For $p_{i1,i2,i3}=\left(\begin{array}{c} ∠(x_{i1},x_{i2})\\ ∠(x_{i1},x_{i3})\\ ∠(x_{i2},x_{i3}) \end{array}\right)$, without loss of generality, derive the bound of its first element $∠\left(x_{i1}+t,x_{i2}+t\right)$, when t falls in a branch T. It turns out to be difficult to calculate a tight angular distance bound, but a simple yet loose bound can be directly obtained from previous studies [43,44].

When a point x is translated by a translation in a cubic branch T, the vector corresponding to the translated point falls in a confined range, which is called the uncertainty angle bound in [43]. The uncertainty angle bound is the maximum deviation of the vector in the range from the one corresponding to point x translated by the center of T. The bound proposed by [43] is directly used in this paper. Given a 3D point x and a cubic translation branch T centered at $t_{0}$ with half space diagonal $\delta_{t}$, then

(7) $$\begin{array}{ll} ∠(x+t_{0},x+t) & \leq \{\begin{array}{c} \arcsin\left(\frac{\delta_{t}}{∥x+t_{0}∥}\right)\;if\;\delta_{t}\leq ∥x+t_{0}∥\\ \pi \;\;\;\;\;\;otherwise \end{array}\\ \; & :=\beta (x,T) \end{array}$$

The proof of (7) can be found in [43].

Thus, the angular distance bound of $∠\left(x_{i1}+t,x_{i2}+t\right)$ when t∈T can be determined directly by using the results of (7):

$$\max(∠\left(x_{i1}+t_{0},x_{i2}+t_{0}\right)-\beta \left(x_{i1},T\right)-\beta \left(x_{i2},T\right),0)$$

(8) $$\begin{array}{l} \leq ∠(x_{i1}+t,x_{i2}+t)\\ \leq \min(∠\left(x_{i1}+t_{0},x_{i2}+t_{0}\right)+\beta \left(x_{i1},T\right)+\beta \left(x_{i2},T\right),\pi ) \end{array}$$

After calculating the bounds of $∠\left(x_{i1}+t,x_{i2}+t\right)$, where t∈T, the second and third elements of $F\left(p^{m\prime },t\right)$ can also be obtained. Then obtain $\underline{D(T)}$ by natural interval extension and the upper bound $\bar{E_{t}\left(T\right)}$ for a translation branch T. By utilizing the upper bound $\bar{E_{t}\left(T\right)}$, we can search the translation space to find the globally optimal translation that maximizes the objective function (4) by BnB. The algorithm is outlined in Algorithm 1.
**Algorithm 1:**Globally Optimal Translation Search Based on RSIFs.
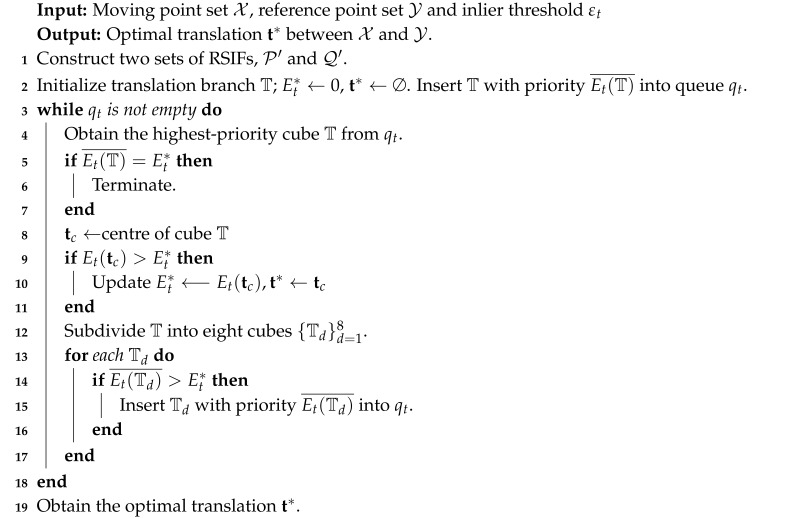


After the global optimal translation $t^{*}$ has been found, the moving point set $X={\left\{x_{i}\right\}}_{i=1}^{X}$ is translated to $X^{*}={\left\{x_{i}^{*}\right\}}_{i=1}^{X}$ where $x_{i}^{*}=x_{i}+t^{*}$. Thus, there are a relative rotation and scale between $X^{*}$ and Y. We first estimate the optimal $R^{*}$.

### 3.3. Similarity Point Set Registration Algorithm

After the global optimal translation $t^{*}$ has been found, another BnB algorithm similar to the rotation search method in [43,44] is used to search the global optimal rotation about the origin. Again, define the objective function on the basis of the cardinality of the inlier set:

(9) $$E_{r}\left(R\right)=\sum_{i}\max_{j}\left\lfloor ∠\left({\mathrm{Rx}}_{i}^{*},y_{j}\right)\leq \varepsilon_{r}\right\rfloor$$

where $\varepsilon_{r}$ is the inlier threshold. Please note that this objective function is not influenced by the relative scale between the two point sets, so the rotation can be calculated without considering the scale factor.

To maximize (9) with BnB, it is required to find an upper bound for a branch of its parameter space. Here, the axis-angle representation of the rotation is used, and then all rotations are contained in a ball of radius π. As in many similar works [24,25], we enclose the ball with a cube with side length 2π as the initial branch of BnB. Given two rotation vectors a and b in the parameter space of rotation and a 3D vector u, it was established in [45] that

(10) $$∠(R_{a}u,R_{b}u)\leq ∥a-b∥$$

where $R_{a}$ and $R_{b}$ are the matrix forms of rotation corresponding to a and b, respectively. Furthermore, given a cube B in the parameter space of rotation, let $v_{p}$ and $v_{q}$ be points at two opposite corners of B. Then, $c:=(v_{p}+v_{q})/2$ is the center of B, and its corresponding rotation matrix is denoted as $R_{c}$. For any rotation a situated in the cube B,

(11) $$\begin{array}{ll} ∠(R_{c}u,R_{a}u) & \leq \min\{∥c-a∥,\pi \}\\ \; & \leq \min\{\frac{∥v_{p}-v_{q}∥}{2},\pi \}\\ \; & :=\mu_{B} \end{array}$$

Then, for ∀R∈B,

(12) $$∠\left({R_{c}x}_{i}^{*},y_{j}\right)-\mu_{B}\leq ∠\left({\mathrm{Rx}}_{i}^{*},y_{j}\right)\leq ∠\left({R_{c}x}_{i}^{*},y_{j}\right)+\mu_{B}$$

Then, the upper bound of the objective function can be defined as

(13) $$\bar{E_{r}\left(B\right)}=\sum_{i}\max_{j}\left\lfloor ∠\left(R_{c}x_{i}^{*},y_{j}\right)\leq \left(\varepsilon_{r}+\mu_{B}\right)\right\rfloor$$

Utilizing the upper bound given by Equation (13), we can search the rotation space to find the globally optimal rotation that maximizes the objective function (9). The algorithm is outlined in Algorithm 2.
**Algorithm 2:**Globally Optimal Rotation Search.
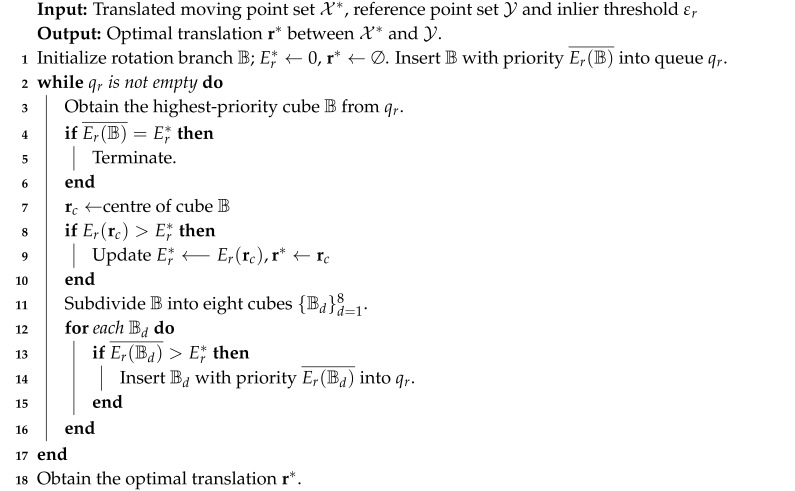


By using Algorithm 2, the global optimal rotation can be obtained. At this point, there is only a scale relationship between the two sets, and the scale can be calculated according to the potential correspondences established in Algorithm 2. Concretely, apply the obtained rotation and translation to the original moving point set, and calculate the angle between any two points of the transformed moving point set and reference point set and the origin. For each point of the moving point set, find the point with the smallest angular distance in the reference point set, which is regarded as the corresponding point. Each pair of corresponding points calculates a scale, and takes the median of these scales as the scale factor estimated by our method.

The full similarity point set registration algorithm is composed of a translation search by Algorithm 1, a rotation search by Algorithm 2, and a final calculation of the scale factor. It should be noted that the decomposed problem is not exactly the same as the original problem formulated in Equation (1), but in practice, it is feasible to find the translation, the rotation and the scale separately.

## 4. Results and Discussion

In this section, we evaluate the performance of our method and compare it against state-of-the-art global and local methods in similarity point set registration. Because there are few new methods dedicated for similarity point set registration, we choose to compare to two recent methods, APM [30] and CPD [23], which can be used for similarity registration. APM and CPD are the state-of-the-art methods for global affine registration and local deformable registration, respectively. In this experiment, we study the runtimes and accuracy of these three methods on synthetic data and clinical data. Our method is implemented in MATLAB R2017b, except the parts of objective function calculation and collinear points screening, which were implemented in C. The code for the comparing methods was obtained from the authors. To avoid excessive runtimes, we set a limit for every method: if it was unable to converge and return a final solution within 1200 s, it will be terminated by force. In the experiment with synthetic data, the ground truth translation, rotation and scale between the moving point set and the reference point set are known. The accuracy of each method is mainly evaluated by success rate, where success means that the angle between the ground truth rotation and the output rotation is less than 0.1 radian and the translation error relative to the ground truth $∥t-t_{gt}∥/∥t_{gt}∥$ is less than 0.1, where t is the estimated translation and $t_{gt}$ is the ground truth translation, and the absolute error between the output scale and the ground truth scale is less than 0.1. All experiments were performed on a laptop with a 2.21 GHz Intel(R) Core(TM) i7-8750H CPU and 8 GB of RAM.

### 4.1. Synthetic Data

**Figure 1:**
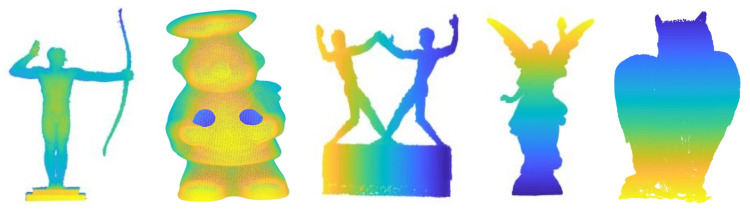
The original 3D models. Archer, Chef, Kids, Lucy and Owl.

We experimented on point sets from four different data sets, which were Chef data set from University of Western Australia [46,47], Lucy data set from the Stanford 3D Scanning Repository [48], Archer and Kids and Owl data sets from EPFL Computer Graphics and Geometry Laboratory [49] and random point data uniformly distributed in ${\left[-1,1\right]}^{3}$. The original 3D models used in this experiment are shown in Figure 1. For our method, the inlier threshold of RSIFs screening was set to 0.01, the inlier threshold of translation search was set to 1, and the inlier threshold of rotation search was set to 10°. The iterations and tolerance error of CPD were set to 10,000 and 1 × $10^{-8}$, respectively. For APM, we used three different tolerance errors: 0.1, 0.2 and 0.3.

#### 4.1.1. Runtime Comparison with APM and CPD

**Figure 2:**
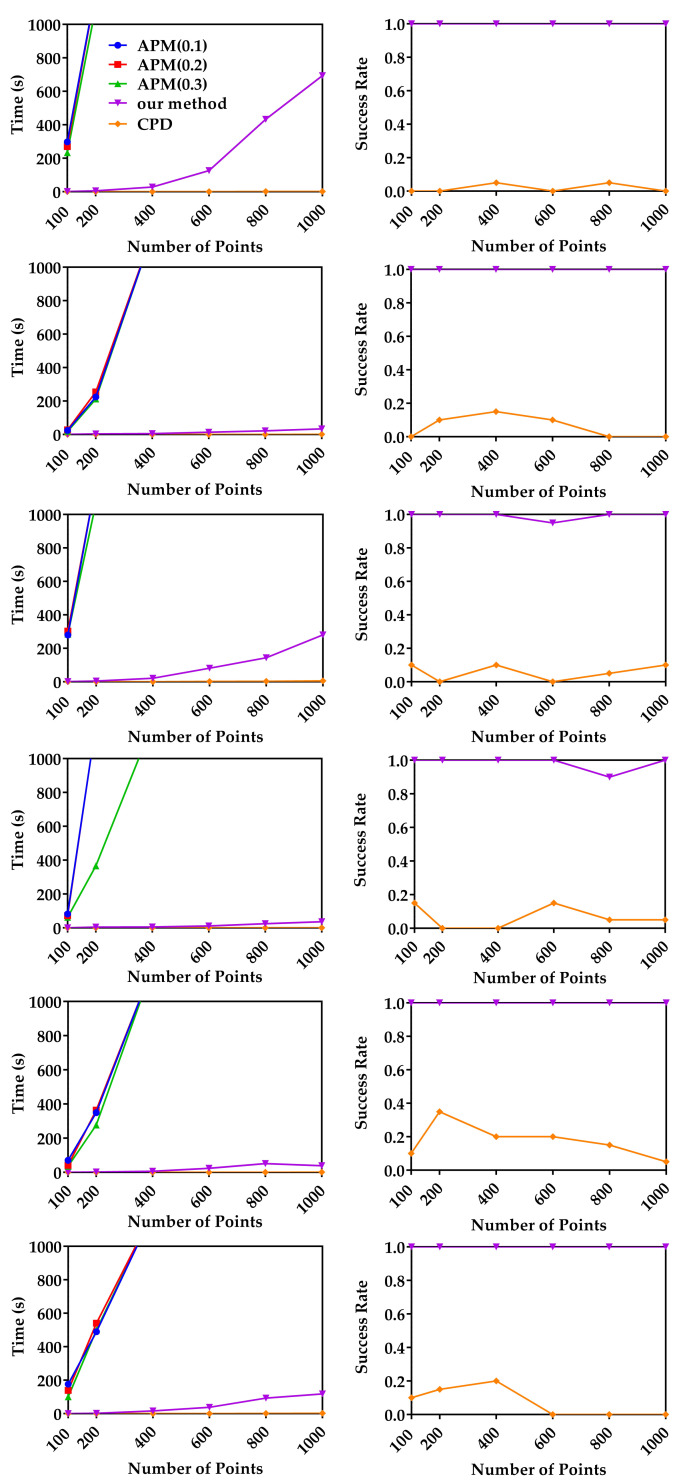
Median runtime and success rate with respective to the number of points. Random data (first row), Archer (2nd row), Chef (3rd row), Kids (4th row), Lucy (5th row) and Owl (last row). Left column: The median runtimes of our method, asymmetric point matching (APM) and coherent point drift (CPD) with respect to the number of points. Right column: The mean success rates of our method and CPD with respect to the number of points.

In this experiment, the runtimes of the three methods with respect to the number of points are studied. The original 3D models were first randomly down-sampled to different numbers of points, and then these points were uniformly scaled to fit in a cube ${\left[-1,1\right]}^{3}$. For random point data, different numbers of uniformly distributed 3D points from ${\left[-1,1\right]}^{3}$ were randomly generated. The down-sampled model data sets and random points were used as moving point sets. For each moving point set, a random similarity transformation (R,t,s) (where 1≤s≤5) was applied to generate a reference point set. For each number of points, we performed 20 registrations with different relative random transformations. Figure 2 shows the median runtime and success rate of APM, CPD and our method with respective to the number of points. We use five models and random data to evaluate our method, APM and CPD. The left column of Figure 2 shows the median runtime of APM, CPD and our method. We set three different tolerance errors, 0.1, 0.2 and 0.3, to APM as denoted APM(0.1), APM(0.2) and APM(0.3). APM exceeded 1200 s in most experiments. On the contrary, as a global method, ours can converge within 1200 s in all experiments. The median runtime of our method on Archer and Kids data sets are less than 40 s, which is 30 times faster than APM. This is because we decouple the translation, rotation, and scale, and we optimize the translation and rotation separately by using two BnB algorithms. The right column of Figure 2 shows the success rate of CPD and our method, and the success rate of APM is not plotted because it cannot terminate in the time limit in most cases. We can see our method achieved 100% success rate on random data (first row), Archer (2nd row), Lucy (5th row) and Owl (last row), although our method failed once in 20 registrations of Chef data with 600 points and twice in 20 registrations of Kids data with 800 points. Meanwhile, the success rate of CPD is very low, because it can only converge to a local optimum. From these results we can see that though the local method CPD is much faster than global methods, its success rate is very low without a proper initialization. Global methods tend to be slow, but our method is much faster than APM.

#### 4.1.2. Robustness to Outliers

**Figure 3:**
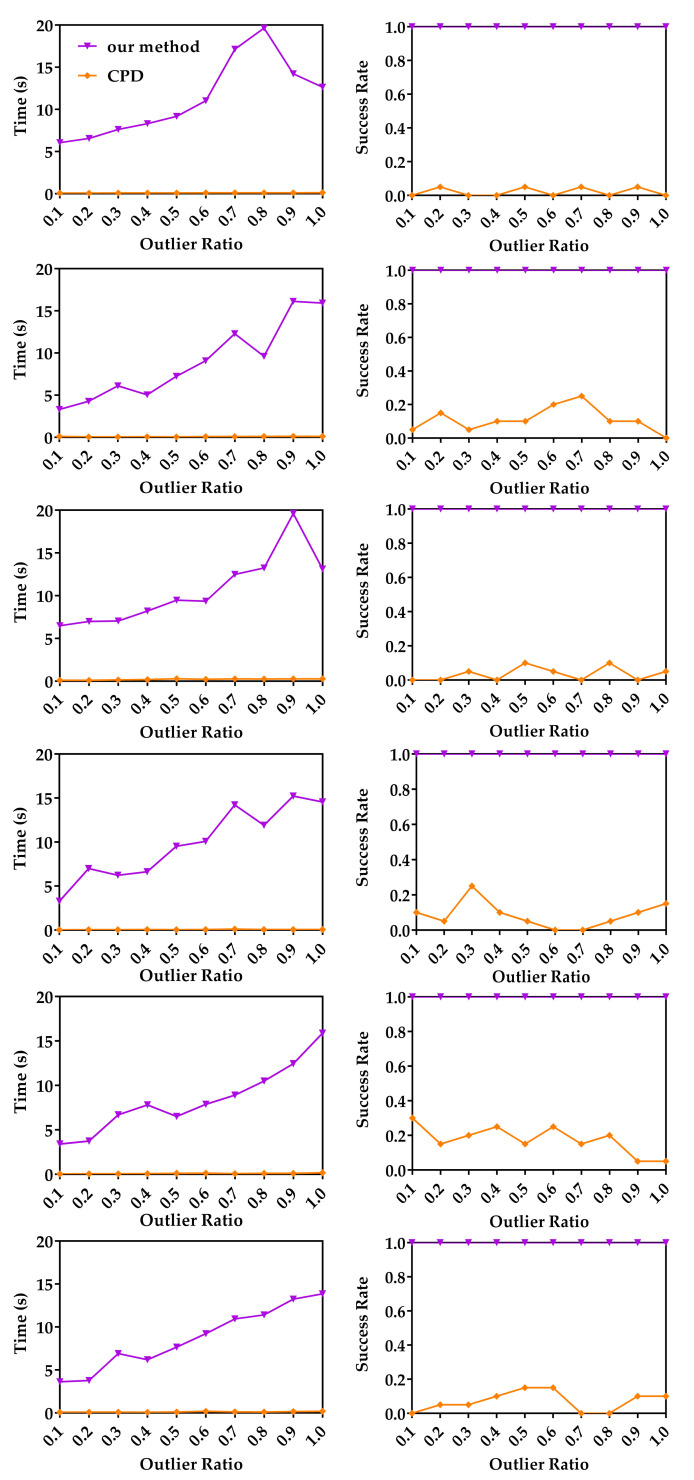
Median runtime and success rate with respective to the outlier ratio. Random data (first row), Archer (2nd row), Chef (3rd row), Kids (4th row), Lucy (5th row) and Owl (last row). Left column: The median runtimes of our method and CPD with respect to the outlier ratio. Right column: The mean success rates of our method and CPD with respect to the outlier ratio.

In this experiment, we compare the robustness of our method, APM and CPD to outliers using the same raw data as in Section 4.1.1. These original model data sets were first randomly down-sampled to 200 points, and then each model was scaled uniformly to make it fit in a cube of ${\left[-1,1\right]}^{3}$. For random point data, 200 points uniformly distributed in ${\left[-1,1\right]}^{3}$ were randomly generated. The down-sampled model data sets and random points were used as moving point sets. For each moving point set, a random similarity transformation (R,t,s) (where 1≤s≤5) was applied and random gross outlier points with different outlier percentages with respect to the inliers were added to obtain the reference point sets. For each outlier ratio, 20 registrations under different random transformations were performed. Figure 3 shows the median runtime and success rate of CPD and our method. Please note that the results of APM are not shown in Figure 3, because APM was unable to terminate within 1200 s in most experiments. The left column of Figure 3 shows the median runtime of CPD and our method. The right column of Figure 3 shows the success rate of CPD and our method. Just as mentioned above, global method APM is very slow, while our method can converge within 20 s in all experiments. Although CPD is fast, it cannot return an accurate result in most cases. Our method achieved 100% success rate, which indicates that our method is very robust to gross outliers. From Figure 3, we can see our method is much faster than the global method APM and more accurate than local method CPD. The reason is that our method uses BnB to optimize the translation and rotation separately, which can guarantee the global optimal, and the decomposition strategy greatly improve the efficiency of BnB.

#### 4.1.3. Robustness to Missing Points

**Figure 4:**
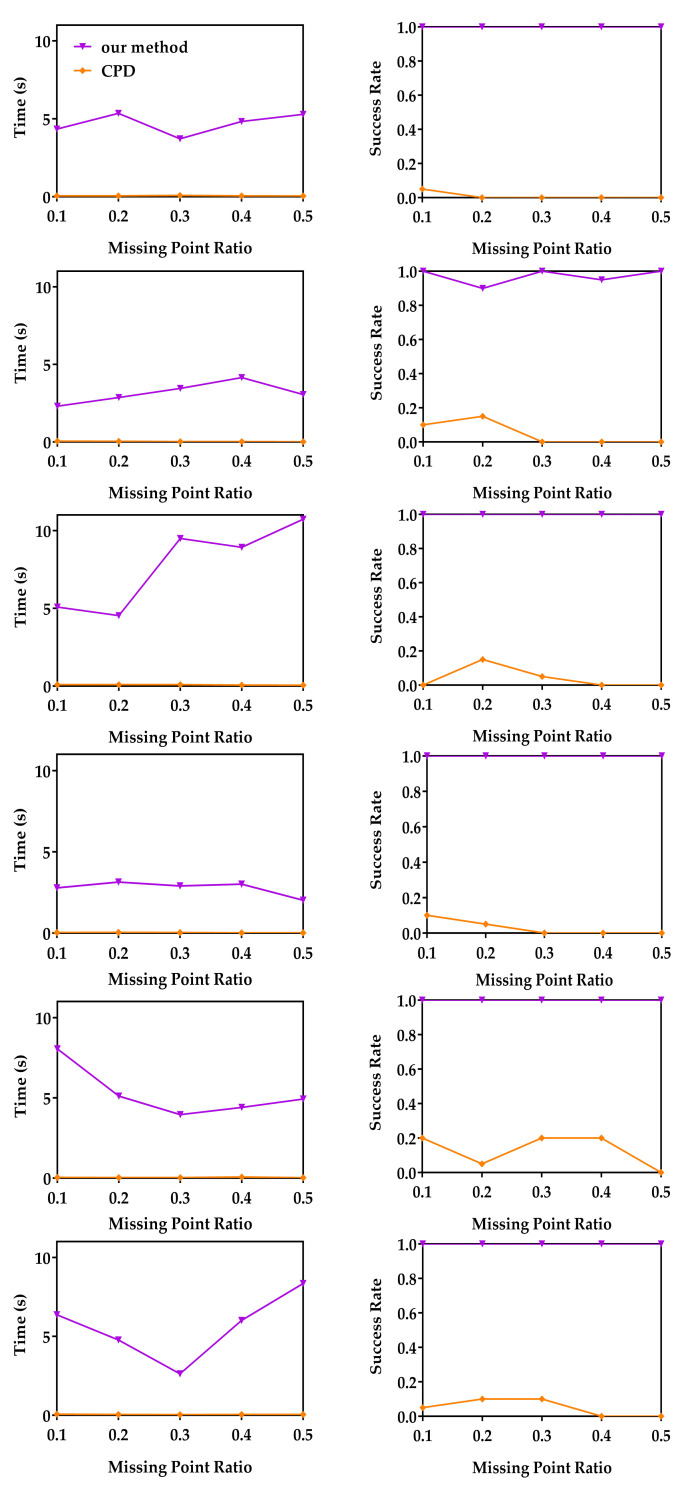
Median runtime and success rate with respective to the missing point ratio. Random data (first row), Archer (2nd row), Chef (3rd row), Kids (4th row), Lucy (5th row) and Owl (last row). Left column: The median runtimes of our method and CPD with respect to the missing point ratio. Right column: The mean success rates of the our method and CPD with respect to the missing point ratio.

In this experiment, we study the robustness of the three methods with respect to the missing point ratio using the same raw data as in Section 4.1.1. These original model data sets were first randomly down-sampled to 200 points, and then each model was scaled uniformly to make it fit in a cube of ${\left[-1,1\right]}^{3}$. For random point data, 200 points uniformly distributed in ${\left[-1,1\right]}^{3}$ were randomly generated. Different ratios of data points were deleted from the down-sampled model data sets and random points to generate the moving point sets, while the reference point sets were constructed by applying random similarity transformation (R,t,s) (where 1≤s≤5) to the entire down-sampled model data sets and random points. For each missing point ratio, 20 registrations under different random transformations were performed. Figure 4 shows the median runtime and success rate of CPD and our method. Please note that the results of APM are not shown in Figure 4, because APM was unable to terminate within 1200 s in most experiments. The left column of Figure 4 shows the median runtime of CPD and our method. The right column of Figure 4 shows the success rate of CPD and our method. From Figure 4, we can see the median runtime of our method is less than 10 s in most cases, which is much faster than the global method APM. The reason may be that the assumption of one-to-one correspondence adopted by APM is not valid in these experiments and transformation decomposition in our method can greatly improve the efficiency of BnB. Our method could successfully register the moving and reference point sets except two failed cases in 20 registrations of the Archer data with 0.2 missing point ratio and one failed case in 20 registrations of the Archer data with 0.4 missing point ratio. Although CPD is fast, it cannot return an accurate result in most cases.

### 4.2. Clinical Data

**Figure 5:**
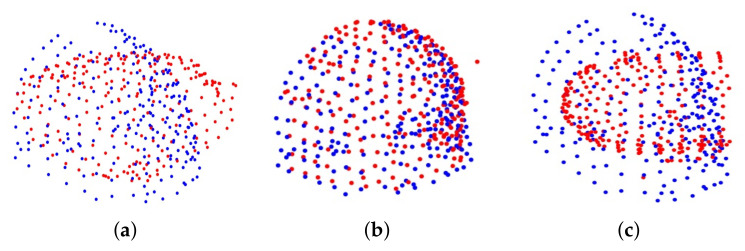
Clinical data before and after registration. (**a**) The initial relative position of the two point sets to be registered. (**b**) The result of our method. (**c**) The result of CPD.

In this section, we compare our method, APM and CPD in registering clinical data. Two scans from 3D MRI volume data and a laser range scanner were used. The point clouds used in these experiments are very dense, and they were down-sampled by using the *pcdownsample* function of MATLAB with a specified *gridsize* 20 to 223 and 241 points, respectively. Then these points were scaled uniformly to make it fit in a cube of ${\left[-1,1\right]}^{3}$. Since there is no ground truth for transformation, in order to calculate the registration error, three targets were selected from the data to calculate the target registration error (TRE). It should be noted that due to the manual selection of the target and the weak correspondence between the data, there is a certain error between the corresponding targets. For our method, the inlier threshold of RSIFs screening was set to 0.1, the inlier threshold of translation search was set to 1, and the inlier threshold of rotation search was set to 10. The iterations and tolerance error of CPD were set to 10,000 and 1 × $10^{-8}$, respectively. For APM, we used three different tolerance errors: 0.1, 0.2 and 0.3. Since APM requires that the number of moving points to be less than the number of reference points, two point sets were exchanged and input into APM. However, APM cannot converge within 1200 s. Then we randomly delete 18 points from the moving point set so that the number of moving points is the same as the number of reference points, but APM still cannot converge in 1200 s. Figure 5 shows the clinical data before and after registration. There is approximately relative rotation of 90 degrees between two point sets in Figure 5a. The result of our method is shown in Figure 5b, and our method could successfully register two point sets. Figure 5c shows the result of CPD. The running time of each method, TRE, mean, and standard deviation are listed in Table 1. The scale of CPD is 0.893, and ours is 1.040. CPD is much faster than our method, but it failed, while our method succeeded in 19 s.

## 5. Conclusions

This paper focuses on the similarity point set registration problem and proposes an efficient global method by using BnB and transformation decomposition. Because the time efficiency of BnB optimization is exponential in the dimensionality of the problem, a novel rotation and scale invariant feature is proposed to decouple the optimization of translation, rotation and scale. The optimal translation is first globally optimized based on the two sets of rotation and scale invariant features constructed from the original point sets. Then, the optimal rotation between the original point sets is calculated after applying the obtained optimal translation to the moving point set. Finally, the scale is estimated through potential correspondences. Decoupling the optimization of the translation, the rotation and the scale makes the proposed algorithm much more efficient than the existing global method. When the outlier ratio with respect to inliers is up to 1.0 or missing point ratio is 0.5, our method still successfully registers the moving point set and reference point set. Extensive experiments show that the proposed method is approximately two orders of magnitude faster than state-of-the-art global method and much more accurate than local methods in similarity point set registration.

There are several limitations of this study. First of all, we have to admit that the decomposed problem is not exactly the same as the original problem. However, in practice, it is feasible to find the translation, the rotation and the scale separately. Secondly, when the outlier ratio is higher, the method may fail. Finally, the application experiment is relatively simple. In the future, we will further optimize the algorithm and enhance its applicability.

Overall, this paper proposes an efficient global similarity point registration algorithm based on transformation decomposition and BnB optimization framework. Two BnB-based algorithms are used to globally estimate translation and rotation in two three-dimensional spaces, which improve the efficiency of BnB. Thus, our method is faster than the state-of-the-art global method. At the same time, our method uses BnB optimization framework to guarantee the global optimality, which makes it more accurate than local method in similarity point set registration.

## Figures and Tables

**Table 1:** The results of registration on the clinical data.

Methods	Time [s]	TRE			Mean	Standard Deviation
		Target No.1	Target No.2	Target No.3		
Our method	18.568	**0.109**	**0.092**	**0.090**	**0.097**	**0.009**
CPD	**0.416**	0.423	0.782	0.472	0.559	0.159
